# A Nanoliposome Platform Co-Delivery of Hydroxypinacolone Retinoate and Carnosine for Enhanced Epidermal/Dermal Delivery and Multi-Functional Anti-Aging Efficacy

**DOI:** 10.3390/pharmaceutics18040454

**Published:** 2026-04-08

**Authors:** Siyuan Chen, Lihao Gu, Ruili Zhao, Lihua Zhang, Lina Yao, Jingning Shen, Dan Luo, Xi Wang, Dan Chen, Si Zhao, Hong Zhou, Wei Liu

**Affiliations:** 1 Research Institute for Biomaterials, Tech Institute for Advanced Materials Bioinspired Biomedical Materials & Devices Center, College of Materials Science and Engineering, Jiangsu Collaborative Innovation Center for Advanced Inorganic Function Composites, Suqian Advanced Materials Industry Technology Innovation Center, Nanjing Tech University, Nanjing 211816, China; siyuan.chen@njtech.edu.cn; 2 Osman Biological Co., Ltd., Huzhou 313200, China; gulihao@osm.com.cn (L.G.); zhaoruili@osm.com.cn (R.Z.); zhanglh@osm.com.cn (L.Z.); yaoln@osm.com.cn (L.Y.); shenjingning@osm.com.cn (J.S.); 3 National Engineering Research Center for Nanomedicine, Huazhong University of Science and Technology, Wuhan 430075, China; laurel565118@163.com; 4 Wuhan Bestcarrier Biotechnology Co., Ltd., Wuhan 430074, China; wx121793@163.com (X.W.); yaoxuechendan@163.com (D.C.); zhaosi0109@163.com (S.Z.); 5 USDA/ARS Children’s Nutrition Research Center, Department of Pediatrics, Baylor College of Medicine, Houston, TX 77030, USA; 6 College of Life Science and Technology, Huazhong University of Science and Technology, Wuhan 430074, China

**Keywords:** Nanoliposomes, co-delivery, hydroxypinacolone retinoate, carnosine, anti-aging, epidermal/dermal delivery

## Abstract

**Background:** Effective anti-aging requires dual strategies to stimulate regeneration and counteract damage. While the combination of hydroxypinacolone retinoate (HPR) and carnosine (CA) holds great promise, their effectiveness is hampered by instability and poor skin penetration. **Methods:** To overcome these challenges, this study developed HPR and CA co-encapsulated nanoliposomes (HC-NLPs) via high-pressure homogenization as an advanced epidermal/dermal delivery system. **Results:** HC-NLPs markedly improved skin retention of HPR (58.97%) and CA (111.36%) compared to the free combination (Free-HC). In cellular studies, HC-NLPs displayed excellent biocompatibility and demonstrated a 4.7-fold higher cellular uptake. This led to enhanced proliferative (EdU positive rate increased by 78.32%) and migratory (wound closure improved by 31.5%) capacities. Moreover, HC-NLPs effectively reinforced multiple skin-protective processes associated with aging, including enhanced resistance to oxidative and glycation-induced damage, suppressed inflammatory responses, and strengthened cellular barrier integrity. In 3D skin models, HC-NLPs promoted collagen deposition and improved tissue morphology compared to Free-HC. Their superior in vivo antioxidant and anti-aging effects were further validated in Zebrafish assays. HC-NLPs effectively co-deliver HPR and CA, markedly improving their stability, skin penetration, and cellular internalization. **Conclusions:** The formulation demonstrates comprehensive pro-regenerative, anti-inflammatory, antioxidative, and anti-glycation effects, representing a promising nano-delivery strategy for advanced anti-aging skincare.

## 1. Introduction

Skin aging is a multifactorial biological process influenced by both intrinsic and chronological factors, as well as extrinsic environmental stressors such as ultraviolet (UV) radiation, pollution, and oxidative stress. These elements collectively contribute to structural and functional deterioration of the skin, manifesting as epidermis thinning, fragmentation of collagen and elastin fibers, and impaired skin barrier function. At the molecular level, the excessive reactive oxygen species (ROS) play a central role by inducing oxidative damage to proteins, lipids, and DNA, further promoting inflammation and cellular senescence. As a result, aged skin exhibits visible changes, including wrinkle formation, loss of elasticity, uneven pigmentation, and delayed wound healing. Conventional anti-aging strategies often focus on a single pathway, for instance, stimulating collagen production with retinoids or providing broad antioxidant support. However, given the complex, multifactorial nature of skin aging, such unilateral approaches often yield limited efficacy. Therefore, a more comprehensive strategy that simultaneously promotes collagen synthesis and protects the extracellular matrix from oxidative and glycative damage may offer superior anti-aging outcomes [1,2,3,4,5].

A promising route to achieving this dual objective is the combination of hydroxypinacolone retinoate (HPR) and carnosine (CA), two actives with complementary mechanisms. HPR, a novel ester derivative of retinoic acid, acts as a potent yet well-tolerated stimulator of collagen production and skin renewal. Unlike conventional retinoids, HPR binds directly to retinoic acid receptors without requiring enzymatic conversion, thereby providing retinoid-like activity with reduced irritation and photosensitivity [6,7]. CA, a naturally occurring dipeptide, serves as a potent antioxidant and antiglycation agent, effectively scavenging ROS and reactive carbonyl species to protect structural proteins such as collagen and elastin from degradation [8,9,10]. Together, HPR and CA constitute a “build and protect” strategy, aiming to both enhance collagen synthesis and shield the extracellular matrix from damage.

Despite their promising profiles, both compounds face significant delivery and stability challenges that limit their topical application. HPR, although more stable than some traditional retinoids, remains susceptible to light, oxygen, and temperature-dependent degradation. Its high lipophilicity also hampers compatibility with aqueous formulations, often necessitating encapsulation strategies to ensure adequate skin penetration. Furthermore, while HPR activates retinoic acid receptors directly, its transcriptional activity may not fully reach that of all-trans retinoic acid, potentially constraining its efficacy in certain contexts [11,12,13]. CA, on the other hand, is highly hydrophilic, which restricts its passive diffusion through the lipophilic stratum corneum. It is also prone to enzymatic degradation and rapid clearance, undermining its stability and bioavailability in topical products. Additionally, formulating CA into stable cosmetic systems while ensuring sufficient skin retention presents technical and economic challenges [14,15].

To overcome these limitations, an advanced delivery system capable of co-encapsulating both hydrophilic and lipophilic actives is required. While various formulation approaches have been explored for anti-aging treatments, such as nanoemulsions, polymeric nanoparticles, and hydrogels, each presents specific constraints when dealing with molecules of opposing polarities [16], e.g., nanoemulsions and lipid nanoparticles are excellent for lipophilic actives such as HPR but often exhibit limited loading capacity and stability for highly hydrophilic peptides like CA [17]. Conversely, hydrogels may fail to provide sufficient skin penetration or a protective lipid environment for unstable retinoids [18]. Liposomes, characterized by their unique amphiphilic architecture consisting of a phospholipid bilayer surrounding an aqueous core, offer an ideal solution. Their bilayer structure allows simultaneous encapsulation of both hydrophilic and lipophilic active ingredients, ensuring a stable and uniform molecular distribution that single-phase systems struggle to achieve [19]. Due to their structural similarity to biological membranes, liposomes exhibit excellent biocompatibility and flexibility for topical and transdermal drug delivery applications [20]. Importantly, liposomes enhance skin penetration by interacting with stratum corneum lipids, provide sustained release of encapsulated actives, and protect sensitive ingredients from environmental and enzymatic degradation [21]. Their nanoscale size and amphiphilic nature also promote uniform distribution on the skin surface and reduce irritation commonly associated with direct application of free actives [22]. These properties, combined with their ability to shield labile compounds from oxidative degradation, make liposomes a uniquely effective platform for coordinated anti-aging therapy. Our research group has successfully developed liposomal platforms to enhance the topical performance of various bioactive peptides and labile actives. For instance, we previously developed copper peptide-, acetyl tetrapeptide-3-, and myristoyl pentapeptide-4-co-loaded nanoliposomes (CAM-NLPs), which displayed uniform nanoscale size, high encapsulation efficiency, and loading capacity. The co-loaded nanoliposomes significantly promoted cellular proliferation, migration, and the secretion of key regenerative factors in vitro, and showed promising pro-regenerative effects in vivo [23].

Building on our previous work with peptide-loaded liposomes, this study addresses the complex challenge of co-delivery of a highly lipophilic retinoid (HPR) and a hydrophilic peptide (CA). Unlike systems carrying similar-polarity payloads, HPR and CA co-loaded nanoliposomes (HC-NLPs) are engineered to bridge extreme solubility gaps and integrate regenerative, anti-glycation, and anti-inflammatory functions into a unified anti-aging platform. The hypothesis driving this research is that nanoliposome encapsulation of this distinct active pair effectively protects them from degradation and facilitates synchronized skin penetration, thereby eliciting superior anti-aging efficacy compared to the free-form combination. Accordingly, the primary objective of this study is to develop and characterize the HC-NLPs system while systematically evaluating its performance through a multi-tiered framework, ranging from 2D cellular senescence assays to 3D reconstructed human skin models and in vivo zebrafish studies. This work highlights the significance of coordinated co-delivery in overcoming formulation barriers and providing a comprehensive strategy for advanced dermocosmetic rejuvenation.

## 2. Materials and Methods

### 2.1. Materials

HPR (Hydroxypinacolone retinoate) was supplied by Shanghai Keqin Technology Co., Ltd. (Shanghai, China), while CA (l-carnosine; β-alanyl-l-histidine) was obtained from Nanjing Leon Biotechnology Co., Ltd. (Nanjing, China). Soybean lecithin (phosphatidylcholine-rich phospholipids) was provided by Shanghai Taiwei Pharmaceutical Co., Ltd. (Shanghai, China). Polyethylene glycol (PEG)-40 hydrogenated castor oil (polyoxyl 40 hydrogenated castor oil, CO40) and coco-glucoside (alkyl polyglucoside surfactant) were purchased from BASF (Ludwigshafen, Germany). Tween 80 (polyoxyethylene (20) sorbitan monooleate, TW-80) was acquired from Aladdin Holdings Group (Beijing, China). Glycerol (propane-1,2,3-triol) and glyceryl tricaprylate/caprate (medium-chain triglycerides, GTCC) were obtained from Kuala Lumpur Kepong Berhad (Ipoh, Malaysia). Cell culture reagents, including Dulbecco’s modified Eagle’s medium (DMEM), fetal bovine serum (FBS), phosphate-buffered saline (PBS, pH 7.4), penicillin–streptomycin solution, and trypsin-EDTA, were purchased from Gibco (Gaithersburg, MD, USA). Hydrogen peroxide (H_2_O_2_) was obtained from Sigma-Aldrich (St. Louis, MO, USA). The Cell Counting Kit-8 (CCK-8) was supplied by Dojindo Laboratories (Kumamoto, Japan). 4′,6-Diamidino-2-phenylindole (DAPI) and Ras Homolog Family Member B (RhoB) were purchased from MedChemExpress (MCE, Monmouth Junction, NJ, USA). Commercial assay kits for reactive oxygen species (ROS), superoxide dismutase (SOD), catalase (CAT), glutathione peroxidase (GSH-Px), and malondialdehyde (MDA) were purchased from Nanjing Jiancheng Bioengineering Institute (Shanghai, China). Enzyme-linked immunosorbent assay (ELISA) kits for matrix metalloproteinase-1 (MMP-1), MMP-3, collagen type I (COL-I), and collagen type IV (COL-IV) were obtained from Beyotime Biotechnology (Shanghai, China).

### 2.2. Preparation and Characterization of HC-NLPs

HC-NLPs were prepared by high-pressure homogenization. Briefly, Phase A was produced by mixing 2.0% (*w*/*w*) HPR, 10.0% (*w*/*w*) GTCC, and 2.0% (*w*/*w*) lecithin. Phase B consisted of 18.0% (*w*/*w*) TW80, 2.0% (*w*/*w*) 1,3-Butylene Glycol, and 20.0% (*w*/*w*) CO40. Phase C was prepared by mixing 1.0% (*w*/*w*) CA, 20.0% (*w*/*w*) glycerol, 2.0% (*w*/*w*) Coco-Glucoside, and 23.0% (*w*/*w*) deionized water. Phase A, Phase B, and Phase C solutions were prepared separately at 45 °C until fully dissolved and clarified. Phase A was combined with Phase B under continuous stirring, followed by the addition of Phase C to obtain a homogeneous mixture. The resulting dispersion was filtered and subsequently processed using a microjet high-pressure homogenizer (AMH-3, Antos Nanotechnology, Suzhou, China) at 800 bar for three cycles. The obtained HC-NLPs were purified by ultrafiltration centrifugation (MWCO 30 kDa, Amicon Ultra, Millipore, Billerica, MA, USA) at 15,000× *g* for 30 min to remove unencapsulated actives. The purified HC-NLPs were used for subsequent characterization and biological evaluation. RhoB-loaded NLPs (RhoB-NLPs) were prepared using the same protocol, with RhoB replacing the active ingredients. HC-NLPs were stored under various conditions, including ambient light, room temperature, 4 °C, −20 °C, and 45 °C. Particle size, polydispersity index (PDI), and HPR content were assessed after 30, 60, and 90 days to evaluate storage stability.

The drug loading efficiency (DLE) and encapsulation efficiency (EE) of HC-NLPs were quantified using an ultrafiltration-centrifugation procedure. Samples were first processed using MWCO 30 kDa filters to separate free drug from the liposomes-encapsulated fraction. The levels of HPR and CA in the resulting filtrates were subsequently determined by high-performance liquid chromatography (HPLC) using a BOCL 101 system (Shimadzu Instruments, Columbia, MD, USA). For HPR analysis, the system was equipped with an Agilent ZORBAX SB-C18 column (4.6 mm × 250 mm, 5.0 µm, Agilent Technologies, Santa Clara, CA, USA). The mobile phase consisted of acetonitrile and water (90:10, *v*/*v*). The chromatographic conditions were: column temperature of 35 °C, UV detection wavelength of 360 nm, injection volume of 10 μL, and flow rate of 1.0 mL/min. The method was validated with a linear range of 6.25–200 µg/mL for HPR (R^2^ = 0.9998). For CA quantification, an HP Amino column (4.6 mm × 250 mm, 5 µm) was employed with a mobile phase consisting of acetonitrile and 30 mM KH_2_PO_4_ buffer (pH 6.8) at a 65:35 (*v*/*v*) ratio. Chromatographic separation was performed at 35 °C with UV detection at 214 nm, an injection volume of 10 µL, and a flow rate of 1.0 mL/min. The calibration for CA exhibited strong linearity between 7.81 and 500 µg/mL (R^2^ = 0.9979). The DLE and EE were calculated using the following equations:

$$\mathrm{DLE}\;\left(\%\right)\;=\frac{W_{e}}{W_{m}}\times \;100$$

$$\mathrm{EE}\;(\%)=\frac{W_{e}}{W_{e}+W_{f}}\times 100$$

where W_e_ denotes the mass of active ingredients encapsulated in the nanocarrier, W_m_ represents the total mass of the nanocarrier, and W_f_ signifies the mass of free, unencapsulated active ingredients.

The physicochemical characteristics of HC-NLPs, namely particle size, polydispersity index (PDI), and zeta potential, were analyzed by dynamic light scattering using a Zetasizer Nano-ZS90 (Malvern Instruments, Malvern, UK). Before measurement, the nanoparticle suspensions were diluted 100-fold with deionized water to ensure optimal scattering conditions. Morphological features of the HC-NLPs were further visualized by transmission electron microscopy (TEM; HT7700, Hitachi, Tokyo, Japan). For TEM analysis, samples were diluted 500-fold, deposited onto copper grids, negatively stained with 1% phosphomolybdic acid, and allowed to air-dry before imaging.

### 2.3. Cell Culture

Human immortalized keratinocyte cell line (HaCaT keratinocytes) and Human dermal fibroblast (HDF) cells (American Type Culture Collection, Manassas, VA, USA) were maintained in DMEM supplemented with 10% FBS and 1% penicillin/streptomycin. The cultures were kept under standard conditions in a humidified incubator at 37 °C with 5% CO_2_ to ensure stable cell growth and viability.

### 2.4. In Vitro Release

The in vitro release profile of HC-NLPs was assessed using a dialysis membrane (MWCO 14 kDa, Yuanye, Shanghai, China). HC-NLPs or Free-HC solution (1 g), which was prepared with the same HPR and CA concentrations and weight ratio as those encapsulated in HC-NLPs using a methanol/water mixture (90:10, *v*/*v*) to ensure complete solubilization of both actives, was added to the dialysis membrane, then immersed in 80 mL of phosphate-buffered saline (PBS solution) containing 15% 1,2-propanediol. This receptor medium was selected to maintain sink conditions for the lipophilic HPR while ensuring the structural integrity of the liposomal phospholipid bilayers throughout the study [24,25,26,27]. In vitro release study was carried out in a shaker (100 rpm, 32°C). At predetermined time intervals (0.5, 1, 2, 4, 6, 8, 10, 12, and 24 h), 1.0 g of the receptor medium was withdrawn and replaced with an equal volume of medium to maintain sink conditions. The cumulative release of HPR and CA was quantified by HPLC as described above. Because the ratio of HPR: CA was fixed across all preparations, the concentrations of both Free-HC and HC-NLP formulations are reported throughout the study on an HPR-equivalent basis.

### 2.5. In Vitro Skin Permeation

Skin permeation was performed using a vertical Franz diffusion cell with excised porcine skin mounted between the donor and receptor chambers. A 5% HC-NLPs formulation (containing 0.1% HPR) or an equivalent Free-HC solution (with an equivalent HPR concentration) was applied to the donor compartment. PBS (pH 7.4) served as the receptor medium to simulate the physiological environment of the dermis, maintained at 32 °C to reflect actual skin surface temperature during permeation.

After the 24 h exposure period, residual formulation on the skin surface was removed by gentle rinsing. The tissue was then finely minced and homogenized, and the resulting mixture was extracted with methanol. Following centrifugation, the clarified supernatant was subjected to HPLC analysis to determine the amounts of HPR and CA retained per unit area of skin.

For visualization of skin permeation, RhoB-NLPs were prepared by substituting the active ingredient with the fluorescent probe RhoB. Excised porcine skin was mounted between the donor and receptor chambers in Franz diffusion cells. Equal amounts of RhoB-NLPs or Free-RhoB solution (equivalent RhoB concentration) were applied to the donor chamber, with PBS as the receptor medium at 32 °C under continuous stirring. After 2 and 4 h, residual formulation on the skin surface was gently removed, and the skin was rinsed, dried, and cryosectioned. Fluorescence distribution across skin layers was observed by confocal laser scanning microscopy (CLSM, IX71, Olympus, Tokyo, Japan) using excitation/emission wavelengths of 564/568 nm.

### 2.6. In Vitro Cytotoxicity

HaCaT and HDF cells in the logarithmic growth phase were harvested by trypsinization, collected by centrifugation, and resuspended in fresh medium. The cell suspensions were then dispensed into 96-well plates at appropriate seeding densities and cultured until reaching approximately 80% confluence. All cells were cultured for 24 h at 37 °C in a humidified atmosphere containing 5% CO_2_. The medium was then replaced with 100 μL of DMEM containing HC-NLPs at HPR-equivalent concentrations of 1.0, 2.0, 4.0, 8.0, or 12.0 μg/mL. Corresponding concentrations of Free-HPR were used as controls, while untreated cells constituted the normal control (NC) group. After 24 h of incubation, cell viability was determined using the CCK-8 reagent according to the manufacturer’s protocol.

### 2.7. Vascular Irritation Assessment Using the HET-CAM Assay

Free-HC or HC-NLPs were diluted tenfold with normal saline to obtain a test sample containing 10% Free-HC or HC-NLPs. Then, a 0.2 mL aliquot of each formulation was gently dispensed onto the chorioallantoic membrane (CAM). Vascular responses were continuously examined over a 5 min period, and the onset of congestion, hemorrhage, and coagulation was documented. The irritation score (IS) was subsequently calculated using the following formula [28]:

IS = [(301 − secH) × 5 + (301 − secL) × 7 + (301 − secC) × 9]/300

In which secH represents the initial time of hyperemia (s), secL represents the initial time of hemorrhage (s), and secC represents the initial time of coagulation (s). The mean value of repeated experiments was calculated, and based on the mean value, the test substance was classified into irritation categories: 0~0.9 (no irritation), 1.0~4.9 (mild irritation), 5.0~8.9 (moderate irritation), and 9~21.0 (severe irritation).

### 2.8. Cellular Uptake Study

HDF cells in the logarithmic growth phase were seeded into 35 mm glass-bottom dishes (3.0 × 10^5^ cells per dish) and cultured for 24 h. Then, the culture medium was replaced with DMEM containing either Free-RhoB or RhoB-NLPs at equivalent RhoB concentrations, followed by incubation for 2 or 4 h. Subsequently, the medium was removed, cells were gently rinsed three times with PBS, fixed with 4% paraformaldehyde, and counterstained with DAPI for 15 min. Intracellular fluorescence distribution was captured using CLSM at excitation wavelengths of 405 nm (DAPI) and 561 nm (RhoB).

For quantitative assessment, HDF cells were seeded in 6-well plates (2.0 × 10^5^ cells per well) and pre-incubated for 24 h. Subsequently, the medium was replaced with DMEM containing either Free-RhoB or RhoB-NLPs at the same fluorescent probe concentration; untreated cells served as the normal control. After 2 and 4 h of exposure, cells were washed with PBS, detached by trypsinization, collected by centrifugation, resuspended in 500 μL PBS, and analyzed by flow cytometry (CytoFLEX, Beckman Coulter, Brea, CA, USA) to quantify fluorescence intensity.

### 2.9. Cellular Proliferation and Migration Assay

The effect on cell proliferation was assessed using a 5-ethynyl-2′-deoxyuridine (EdU) assay. After incubating HDF cells to an appropriate density, the medium was substituted with Free-HC or HC-NLPs at an equivalent HPR concentration (4 μg/mL). Cells cultured in DMEM alone served as the NC group. After 48 h of incubation, each sample was processed using the BeyoClick EdU Cell Proliferation Kit following the manufacturer’s protocol. The nuclei were counterstained with DAPI (2 μg/mL) for 10 min, and fluorescence signals were subsequently visualized by CLSM. EdU-positive cells were detected using the 495/519 nm excitation/emission settings for Azide 488.

For the cell viability assessment, HDF cells were seeded in 96-well plates at a density of 8 × 10^3^ cells per well. After cell attachment, each well received 100 µL of complete DMEM containing HC-NLPs at HPR-equivalent concentrations of 1.0, 2.0, or 4.0 µg/mL. Corresponding Free-HC groups (matching the active ingredient concentration of the HC-NLPs) and a DMEM-only control (NC) were included. After 48 h of incubation, cell viability was quantified using the CCK-8 assay, with each treatment performed in triplicate.

For the scratch wound healing assay, HDF cells were seeded into 6-well plates and allowed to grow until reaching approximately 90% confluence. A linear wound was created in the confluent monolayer using a sterile 200 μL pipette tip. Detached cells were removed by gentle PBS rinsing. Cells were then treated with Free-HC or HC-NLPs (both at 4 μg/mL HPR-equivalent) for 24 h. Microscopic images of the scratch area were acquired at 0 h and 24 h using an inverted microscope, and the extent of wound closure was quantified with ImageJ. 1.8.0.

### 2.10. Assessment of Intracellular β-Galactosidase Inhibition and Anti-Glycation Activities

HDF cells in the logarithmic growth phase were seeded in 6-well plates at a density of 1.5 × 10^5^ cells per well and incubated overnight. Four experimental groups were established: normal control (NC), model control (MC, treated with d-galactose), Free-HC, and HC-NLPs, with three replicates per group. Subsequently, the corresponding treatments were added and co-incubated with d-galactose for 48 h (the NC group received DMEM only, while the MC group received d-galactose in DMEM). After incubation, cells were washed with PBS, fixed with 1 mL of β-galactosidase staining fixative for 15 min at room temperature, and then stained according to the manufacturer’s protocol (β-galactosidase assay kit. Finally, the stained HDF cells were observed and imaged using an inverted fluorescence microscope to assess the effects of Free-HC and HC-NLPs on d-galactose-induced cellular senescence [28].

HDF cells were seeded at the same density and assigned to groups as previously described. Cells in the MC, Free-HC, and HC-NLPs groups were exposed to 50 mmol/L glucose to induce glycation-mediated cellular aging and simultaneously treated with the respective samples for 72 h [29]. At the end of incubation, glycation-associated senescence indicators were measured using ELISA kits and real-time qPCR. The relevant primer sequences are shown in Table 1.

### 2.11. Cellular Antioxidant Activity Study

HDF cells were seeded in 24-well plates at 4 × 10^4^ cells per well. To induce oxidative stress, cells in the MC, Free-HC, and HC-NLPs groups were treated with DMEM containing 0.6 mM H_2_O_2_; the Free-HC and HC-NLPs groups additionally received Free-HC or HC-NLPs treatment containing 4 μg/mL HPR. The NC group was maintained in fresh DMEM without H_2_O_2_. After 24 h of incubation, cell culture supernatants were collected, and the levels of superoxide dismutase (SOD), malondialdehyde (MDA), catalase (CAT), and glutathione peroxidase (GSH-Px) were measured using commercial ELISA kits. For intracellular ROS assessment, the cells were incubated with DCFH-DA (2′,7′-dichlorofluorescin diacetate, 20 μM in DMEM) for 20 min to allow probe uptake and oxidation. Afterward, they were washed thoroughly with PBS to remove excess dye and immediately examined by confocal laser scanning microscopy using the 488/525 nm excitation-emission settings. The fluorescence intensity of each sample was subsequently quantified by flow cytometry to provide a complementary measure of intracellular ROS levels.

### 2.12. Cellular Anti-Inflammatory Study

HDF cells in the logarithmic growth phase were seeded in 12-well plates at 2 × 10^5^ cells/mL and incubated overnight. Cells were divided into five groups: NC, MC (stimulated with Tumor Necrosis Factor-alpha (TNF-α) and Interferon-gamma (IFN-γ), each at 10 ng/mL), Free-HC, and HC-NLPs. Except for the NC group, all groups were exposed to TNF-α/IFN-γ for 24 h to induce an inflammatory response mimicking skin allergy. Free-HC and HC-NLPs groups were additionally treated with Free-HC or HC-NLPs at 1.0, 2.0, or 4.0 μg/mL. After treatment, culture supernatants were harvested, and the concentrations of interleukin (IL)-1α, TNF-α, IL-6, and IL-8 were quantified using commercial ELISA kits (Jiangsu Meimian Industrial Co., Ltd., Yancheng, China) [30].

### 2.13. Anti-Aging Factors Secretion Level Detection

HDF cells were seeded in 24-well plates at 4 × 10^4^ cells per well and incubated for 24 h. Subsequently, the cells were exposed to HC-NLPs or Free-HC at HPR concentrations of 1.0, 2.0, or 4.0 μg/mL. The NC and MC groups received complete DMEM only, whereas the positive control (PC) group was treated with 40 μg/mL vitamin C (VC). After 1 h of incubation, all groups except the NC group were exposed to UVA irradiation (Philips, Amsterdam, The Netherlands, 200,000 J/m^2^). Following an additional 24 h incubation, the supernatant was collected, and the levels of matrix metalloproteinase (MMP)-1, MMP-3, collagen I, and collagen IV were determined using ELISA kits.

### 2.14. Three-Dimensional Skin Model Anti-Aging Study

Anti-aging efficacy was evaluated using a commercial ex vivo human skin model (Biocell Biotechnology Co., Ltd., Dongguan, China) that retains the full histological structure of native skin (epidermis, dermis, and stratum corneum). Before treatment, tissue samples were equilibrated overnight in 6-well plates under standard culture conditions (37 °C, 5% CO_2_, ~95% relative humidity), and randomly assigned to four groups: NC, MC, PC, Free-HC, and HC-NLPs (the latter containing 200 μg/mL HPR-equivalent). All groups except the NC were irradiated with UVA (300,000 J/m^2^) and UVB (Philips, 500 mJ/m^2^) irradiation, while the NC group remained untreated as the normal control. The PC group was subsequently treated with 100 μg/mL VC and 7 μg/mL vitamin E (VE) following UV exposure. After 24 h of incubation, samples were fixed in 4% paraformaldehyde for 24 h. Expression of collagen I and IV was visualized by immunofluorescence using a fluorescence microscope (excitation/emission: 488/518 nm).

### 2.15. Antioxidant and Whitening Effect Evaluation in Zebrafish

Adult zebrafish (*Danio rerio*) were maintained under standard laboratory conditions with controlled temperature, pH, and light-dark cycles. Before experimentation, fish were acclimated for two weeks with continuous water filtration. Oxidative stress was induced in wild-type zebrafish embryos using a hydroxyacetone model.

Wild-type zebrafish embryos were pretreated with phenylthiourea (PTU, 0.03 mg/mL) to suppress melanogenesis and maintained until 72 h post-fertilization. Embryos were then allocated into 24-well plates (10 embryos per well, three replicates per group). The experimental groups included NC (Holtfreter’s buffer), MC (0.025 g/L hydroxyacetone), PC (0.025 g/L hydroxyacetone plus 0.1 g/L glutathione), Free-HC, and HC-NLPs, with the latter two groups receiving hydroxyacetone and an HPR-equivalent concentration of 200 μg/mL. After incubation at 28 ± 1 °C in the dark for 2 h, embryos were washed with Holtfreter’s buffer and stained with H2DCFDA (5 μM) for 1 h in the dark. Following washing and a 30 min equilibration period, intracellular ROS levels were visualized using a fluorescence microscope (excitation/emission: 488/525 nm).

Zebrafish embryos at 24 hpf were distributed into 24-well plates (10 embryos per well, three replicates per group). Embryos were exposed to Holtfreter’s buffer (NC group) or treated with Free-HC or HC-NLPs at an HPR-equivalent concentration of 200 μg/mL for 48 h. After treatment, embryos were washed, collected, and subjected to RNA extraction. The mRNA expression levels of Elastin (Elna), collagen type I alpha 1 chain (Col1a1), and collagen type I alpha 2 chain (Col1a2) were subsequently quantified by qPCR.

### 2.16. Statistical Analysis

All results are shown as mean ± SD from at least three independent experiments. Statistical analysis was performed with one-way ANOVA. *p* < 0.05 was considered statistically significant.

## 3. Results

### 3.1. Characterization of HC-NLPs

**Figure 1:**
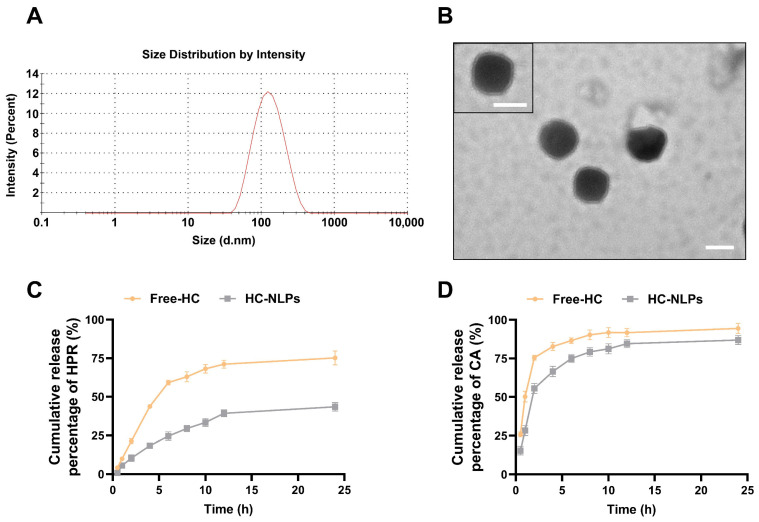
Characterization of HC-NLPs. (**A**) TEM image of HC-NLPs. (**B**) particle size distribution of the HC-NLPs measured by DLS. scale bar: 100 nm. In vitro release profiles of (**C**) HPR and (**D**) CA from Free-HC and HC-NLPs over 24 h. Mean ± SD (*n* = 3).

The prepared HC-NLPs appeared as a transparent, pale yellow liquid. DLS analysis revealed a mean particle size of 111.7 nm (Figure 1A), a low PDI of 0.117, and a zeta potential of −34.5 mV. Transmission electron microscopy (TEM) images (Figure 1B) confirmed a spherical morphology with distinct bilayer structures and a uniform size distribution. The vesicles were well-dispersed without aggregation, indicating good colloidal stability. The relatively small particle size and narrow size distribution indicate the formation of a homogeneous nanosystem, which is generally favorable for topical delivery, as smaller vesicles can facilitate closer interaction with the stratum corneum. The negative zeta potential suggests good electrostatic repulsion between vesicles, contributing to stability [31]. The observed particle size was consistent with DLS measurements. Under the optimized formulation conditions, the EE of HPR and CA was 97.3 ± 0.8% and 71.4 ± 0.4%, respectively, with DLE of 1.60 ± 0.3% and 0.70 ± 0.1% (Table 2). The higher EE observed for HPR is attributed to its lipophilic nature, which favors incorporation into the phospholipid bilayer, whereas the relatively lower EE of CA is likely due to its hydrophilic character. These results demonstrate the capability of liposomes to simultaneously encapsulate compounds with different physicochemical properties. Quality control data are summarized in the Supplementary Materials (Tables S1 and S2). No significant changes in particle size, PDI, or HPR content were observed over time, demonstrating the good stability of the formulation. While its physical stability under storage conditions has been confirmed, the photostability of the HC-NLP system requires further quantitative investigation. Future work will therefore focus on systematic photostability testing under simulated solar radiation to optimize the formulation for daytime or outdoor applications.

The in vitro release profiles of Free-HC and HC-NLPs were evaluated (Figure 1C,D). HC-NLPs exhibited sustained-release characteristics compared to the free formulation. After 12 h, the cumulative release of HPR and CA from HC-NLPs was 71.07% and 39.26%, respectively, significantly lower than that from Free-HC (91.76% and 84.59%). The sustained-release profile is primarily governed by the lipid bilayer barrier, which regulates the diffusion of encapsulated molecules. This controlled release is advantageous for maintaining a consistent therapeutic concentration on the skin and prolonging the residence time of actives, which is beneficial for enhancing efficacy [32].

### 3.2. In Vitro Skin Permeation

**Figure 2:**
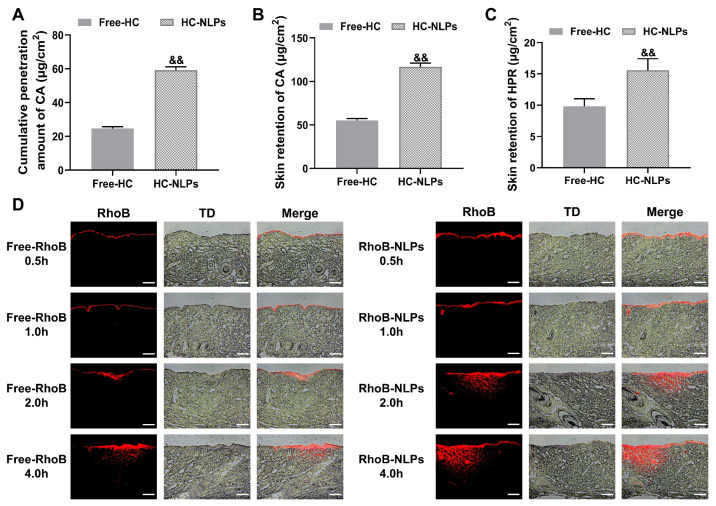
In vitro skin permeability of Free-HC and HC-NLPs. (**A**) Cumulative skin permeation and (**B**) retention of CA over 24 h. (**C**) Cumulative skin retention of HPR. ^&&^ *p* < 0.01 vs. Free-HC. Mean ± SD, *n* = 3. (**D**) Fluorescence microscopy images showing the penetration of RhoB (red) in porcine skin over 4 h. Scale bar: 200 μm.

One major limitation of conventional formulations is the insufficient cutaneous retention of active ingredients. To address this issue, HPR and CA were encapsulated into HC-NLPs to improve their skin delivery efficiency. Franz diffusion cell experiments demonstrated that HC-NLPs markedly enhanced bioavailability. Notably, HPR was not detected in the receptor medium during the 24 h study, indicating minimal transdermal permeation and low risk of systemic absorption [33,34]. HPLC quantification showed that, compared with Free-HC, HC-NLPs increased the skin retention of HPR and CA by 58.97% and 111.36%, respectively (Figure 2B,C). This enhanced retention can be attributed to the affinity of liposomal vesicles for the stratum corneum and their ability to form a depot within the skin [35]. These results indicate that the NLP-based delivery system facilitates greater intradermal accumulation of the hydrophobic actives.

Skin penetration was further visualized using RhoB as a fluorescent probe (Figure 2D). At 0.5 h, signals from both formulations were confined to the stratum corneum. By 1 h, Free-RhoB remained superficial, whereas RhoB-NLPs began migrating into deeper epidermal layers. After 2 h, RhoB-NLPs reached a depth of 448 μm (penetrating into the dermis), significantly deeper than Free-RhoB (101 μm). This difference was further pronounced at 4 h, with RhoB-NLPs extending to 682 μm compared to 457 μm for Free-RhoB. These findings confirm that the NLP system significantly enhances both penetration rate and depth (*p* < 0.05, Figure S1). The improved penetration stems from the nanoscale size of the liposomes and their lipid composition, which can enhance interaction with the stratum corneum lipids and promote transport across the skin barrier. Such enhanced delivery to deeper skin layers is particularly relevant for anti-aging applications, as dermal fibroblasts and extracellular matrix components are primarily located in the dermis. Notably, while previous reports on HPR-loaded nanoemulsions indicated a skin retention of approximately 6 μg/cm^2^ [36], the HC-NLP system achieved a significantly higher retention of 15 μg/cm^2^. This 2.5-fold increase highlights the superior dermal delivery efficiency of the liposomal carrier over conventional emulsions, providing a more potent strategy for anti-aging therapy [37,38,39].

### 3.3. In Vitro Safety Evaluation

In vitro cytotoxicity profiles of Free-HC and HC-NLPs at varying concentrations against HDF and HaCaT cells are presented in Figure 2A,B. At HPR concentrations of 1.0–4.0 μg/mL, both Free-HC and HC-NLPs maintained cell viability above 95% in both cell lines, indicating no cytotoxicity. At HPR concentration of 8 μg/mL, both Free-HC and HC-NLPs exhibited a certain degree of cytotoxicity toward HaCaT cells, with inhibition rates of 7.47% and 5.80%, respectively. When the HPR concentration reached 12 μg/mL, Free-HC showed a 16.34% inhibition of HaCaT cells, in contrast to the 6.45% inhibition observed with HC-NLPs. Under the same conditions, only the Free-HC group showed a significant inhibitory effect on HDF cells, with an inhibition rate of 6.94% (*p* < 0.05). The reduced cytotoxicity observed with HC-NLPs suggests that encapsulating active compounds within the liposomal structure helps modulate their release, limiting direct exposure of cells to high concentrations. This controlled delivery may reduce acute cellular stress, contributing to improved biocompatibility [40].

**Figure 3:**
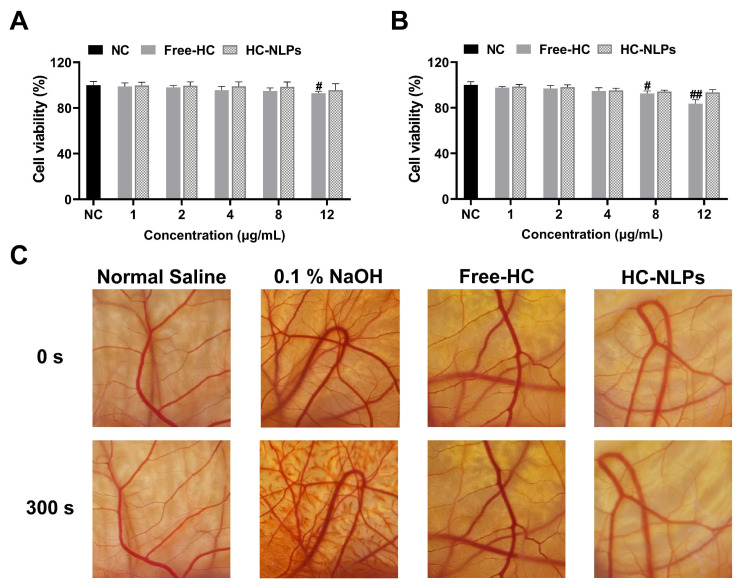
In vitro safety evaluation of Free-HC and HC-NLPs. Cytotoxicity toward (**A**) HDF and (**B**) HaCaT cells after exposure to Free-HC and HC-NLPs at equivalent HPR concentrations (1.0 to 12.0 µg/mL), assessed by the CCK-8 assay. ^#^ *p* < 0.05, ^##^ *p* < 0.01 vs. NC. Mean ± SD, *n* = 5. (**C**) Representative images from the HET-CAM irritation assay after treatment with Free-HC or HC-NLPs at 10% concentration (corresponding to 0.2% HPR).

The biocompatibility of HC-NLPs was further examined. As presented in Figure 3C and Table S2, neither Free-HC nor HC-NLPs elicited detectable hemolysis or vascular irritation under the experimental conditions, with both formulations producing irritation scores that remained essentially negligible. These results confirm the absence of irritancy and highlight the favorable safety profile of the prepared NLPs system.

### 3.4. Cellular Uptake 

**Figure 4:**
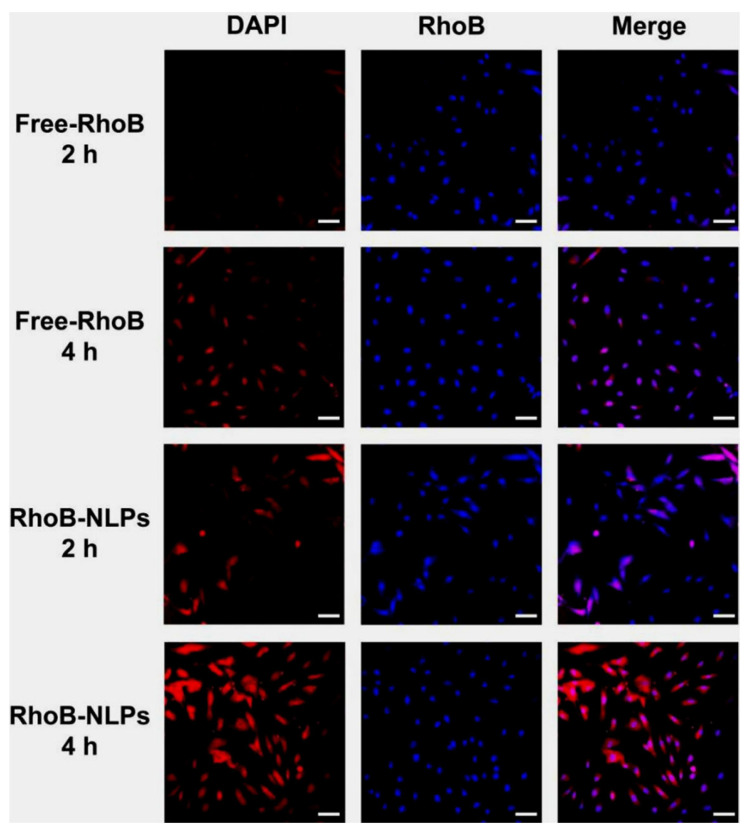
Cellular uptake of Free-RhoB and RhoB-NLPs in HDF cells. CLSM images showing intracellular RhoB fluorescence after 2 h and 4 h incubation. Scale bar: 50 μm.

Cellular internalization of the formulations was evaluated by fluorescence microscopy using RhoB as a fluorescent probe. As illustrated in Figure 4, only faint intracellular fluorescence was observed in HDF cells exposed to Free-RhoB after 2 h of incubation. In contrast, cells treated with RhoB-NLPs displayed stronger red fluorescence at an equivalent RhoB concentration, indicating enhanced uptake. With prolonged incubation, intracellular fluorescence intensity progressively increased. After 4 h, the RhoB-NLPs group exhibited a further pronounced enhancement in fluorescence signal, suggesting sustained cellular internalization. Quantitative analysis by flow cytometry corroborated these observations (Figure S2), revealing that the mean fluorescence intensity of HDF cells treated with RhoB-NLPs was 1.70-fold and 2.04-fold higher than that of the Free-RhoB group at 2 h and 4 h, respectively (*p* < 0.01). The increased uptake of RhoB-NLPs is likely due to the nanoscale size and lipid-based composition of the liposomes, which enhance their interaction with the cell membrane and support endocytic internalization. Additionally, the vesicular structure may improve the intracellular delivery and retention of the encapsulated cargos [40].

Collectively, these findings demonstrate that nanoliposomal encapsulation markedly promotes cellular uptake and intracellular accumulation of the incorporated payload in HDF cells, thereby improving delivery efficiency to target skin cells.

### 3.5. Cell Proliferation and Migration

**Figure 5:**
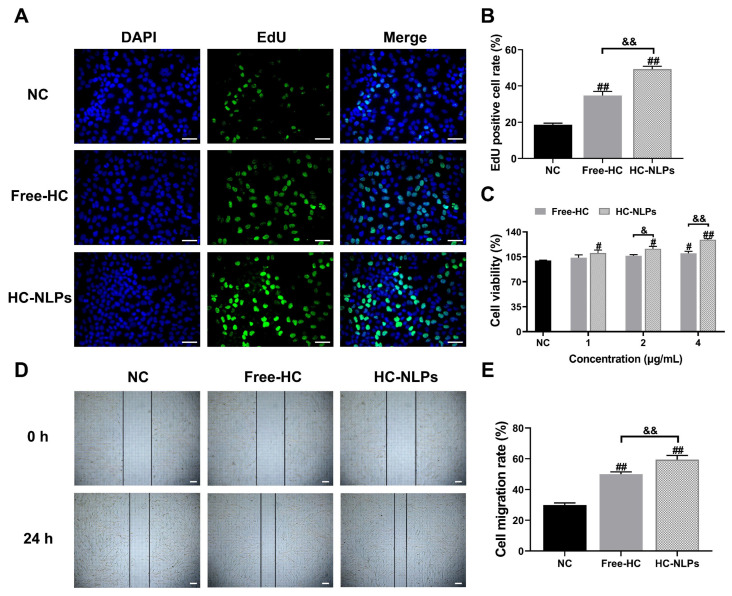
Effects of Free-HC and HC-NLPs on cell proliferation and migration. (**A**) Representative EdU staining images (EdU: green, nuclei: blue; scale bar: 50 μm). (**B**) Quantification of EdU-positive cell rate after 24 h co-incubation. (**C**) Cell proliferation rate of HDF cells after 24 h treatment with Free-HC or HC-NLPs, measured by the CCK-8 assay. (**D**) Microscopic images of scratch wounds at 0 h and 24 h (scale bar: 200 μm) and (**E**) corresponding cell migration rate after 24 h treatment with Free-HC or HC-NLPs. ^#^ *p* < 0.05, ^##^ *p* < 0.01 vs. NC; ^&^ *p* < 0.05, ^&&^ *p* < 0.01. Mean ± SD, *n* = 3.

The EdU assay revealed that both Free-HC and HC-NLPs significantly promoted HDF cell proliferation after 24 h compared to the NC group (*p* < 0.05). The proportion of EdU-positive cells increased by 87.12% and 165.35% in the Free-HC and HC-NLPs groups, respectively. Consistently, the CCK-8 assay results (Figure 5C) demonstrated that HC-NLPs promoted cell proliferation at all tested concentrations. At a HPR concentration of 4 μg/mL, both Free-HC and HC-NLPs enhanced cell viability, reaching 109.23% and 129.02%, respectively.

Furthermore, as shown in Figure 5D,E, after 24 h of treatment with Free-HC or HC-NLPs, the wound areas exhibited varying degrees of closure. Compared with the NC group, the wound-healing rate increased by 67.28% in the Free-HC group and by 98.78% in the HC-NLPs group. The increased proliferation and migration observed with HC-NLPs may be due to the enhanced cellular delivery of the encapsulated actives, which allows them to interact with intracellular targets involved in cell growth and repair processes [41]. Collectively, these findings indicate that both Free-HC and HC-NLPs effectively promote cell proliferation and migration, with HC-NLPs exhibiting superior efficacy.

### 3.6. Inhibition of Cellular Senescence and Advanced Glycation End Products

**Figure 6:**
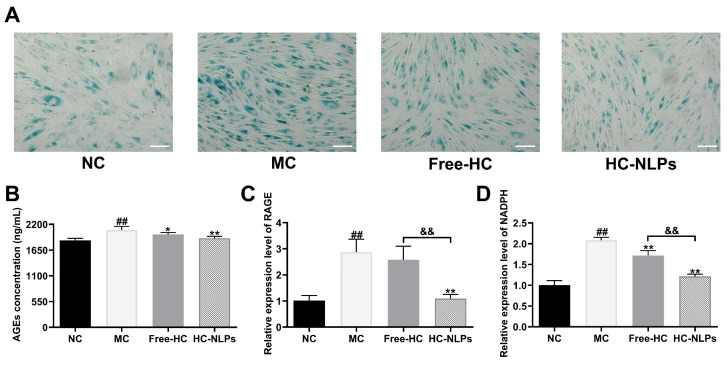
Anti-senescence and anti-glycation effects. (**A**) β-galactosidase staining images showing d-galactose-induced senescence in HDF cells and its attenuation by treatment with Free-HC or HC-NLPs. Scale bar: 50 μm. HDF cells were induced to glycation aging by high-glucose stimulation, followed by treatment with Free-HC or HC-NLPs, and (**B**) AGEs concentration as well as the gene expression levels of (**C**) RAGE and (**D**) NADPDH were measured. ^##^ *p* < 0.01 vs. NC; * *p* < 0.05, ** *p* < 0.01 vs. MC; ^&&^ *p* < 0.01. Mean ± SD, *n* = 3.

As shown in Figure 6A, d-galactose stimulation markedly increased β-galactosidase activity in HDF cells, as evidenced by a substantial rise in positively stained senescent cells in the MC group compared with the NC group. Treatment with Free-HC partially alleviated d-galactose-induced cellular senescence, showing a noticeable reduction in β-galactose-positive cells. Notably, cells treated with HC-NLPs exhibited the lowest level of β-galactosidase staining among all d-galactose-treated groups, indicating a more pronounced suppression of senescence-associated β-galactosidase activity. These findings suggest that both Free-HC and HC-NLPs mitigate d-galactose-induced senescence in HDF cells, with HC-NLPs demonstrating superior anti-aging efficacy.

Under hyperglycemic or chronic metabolic stress conditions, non-enzymatic glycation of proteins and lipids leads to the accumulation of advanced glycation end products (AGEs), which contribute to aging-related tissue dysfunction and impaired cellular homeostasis. AGEs interact with their primary receptor, receptor for advanced glycation end products (RAGE), triggering intracellular signaling cascades that exacerbate oxidative stress and inflammation. This AGE-RAGE engagement activates downstream pathways such as nuclear factor-kappa B (NF-κB) and mitogen-activated protein kinase (MAPK), leading to the induction of pro-inflammatory cytokines and upregulation of nicotinamide adenine dinucleotide phosphate (NADPH) oxidase (NOX) complexes, a major source of ROS in many cell types. NOX activation further amplifies intracellular ROS generation, promoting oxidative damage, redox imbalance, and cellular dysfunction in aging and disease. Importantly, recent evidence indicates that RAGE activation not only increases NOX-derived ROS but also upregulates RAGE expression itself, thereby establishing a positive feedback loop that perpetuates oxidative stress and AGE accumulation. These mechanisms support the central role of the AGEs-RAGE-NADPH oxidase axis in glycation-associated aging and related pathologies [42,43,44,45,46].

Figure 6B–D shows that high-glucose exposure markedly induced glycation-associated cellular senescence in HDF cells. Compared with the NC group, cells in the MC group exhibited a significant increase in intracellular AGEs accumulation, accompanied by pronounced upregulation of RAGE and NADPH oxidase-related gene expression, confirming successful establishment of a glycation-mediated aging model. Treatment with both Free-HC and HC-NLPs significantly attenuated high-glucose-induced glycation stress. ELISA analysis demonstrated that intracellular AGEs levels were markedly reduced in both treatment groups compared with the MC group (*p* < 0.05 or *p* < 0.01). Notably, HC-NLPs achieved a more pronounced reduction in AGEs accumulation than Free-HC. Consistently, real-time qPCR analysis revealed that high-glucose-induced upregulation of RAGE and NADPH oxidase-related genes was significantly suppressed following treatment with Free-HC and HC-NLPs. Importantly, HC-NLPs exhibited significantly stronger inhibitory effects on the expression of both RAGE and NADPH compared with free HC, indicating superior efficacy in mitigating glycation-associated oxidative signaling. Similar improvements in bioactivity have been reported for nanocarrier-based systems, where enhanced cellular uptake and stability contribute to better biological performance [47,48].

Collectively, these results demonstrate that HC effectively alleviates glycation-induced cellular aging in HDF cells, and that nano-liposomal encapsulation substantially enhances its anti-glycation and anti-oxidative efficacy under high-glucose conditions.

### 3.7. Cellular Antioxidant Activity

ROS, chemically reactive derivatives of oxygen generated during cellular metabolism, initiate oxidative stress when excessively accumulated. This leads to lipid peroxidation, protein damage, and cellular dysfunction, which are key events involved in skin aging. Major enzymatic antioxidants, including SOD and CAT, act cooperatively to neutralize ROS: SOD catalyzes the conversion of superoxide anions into hydrogen peroxide, while CAT subsequently decomposes hydrogen peroxide into water and oxygen. GSH-Px represents another crucial antioxidant enzyme that reduces hydrogen peroxide and lipid peroxides using reduced glutathione as an electron donor. MDA, a stable end-product of lipid peroxidation, serves as a biomarker of oxidative damage. Collectively, the balance among SOD, CAT, and GSH-Px activities, as well as the cellular MDA content and ROS levels, reflects the intracellular antioxidant defense status [2,49,50,51,52].

**Figure 7:**
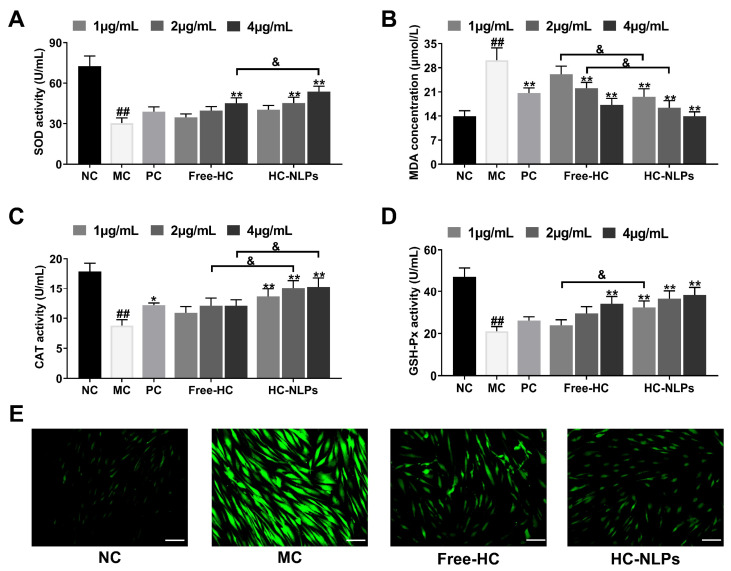
Antioxidant effects of Free-HC and HC-NLPs in HDF cells. Levels of (**A**) SOD, (**B**) MDA, (**C**) CAT, and (**D**) GSH-Px in the supernatant of HDF cells after 24 h of co-incubation with H_2_O_2_ and Free-HC or HC-NLPs. (**E**) Intracellular ROS fluorescence images were observed by CLSM after staining with a ROS detection kit (Scale bar: 50 μm). ^##^ *p* < 0.01 vs. NC; * *p* < 0.05, ** *p* < 0.01 vs. MC; ^&^ *p* < 0.05. Mean ± SD, *n* = 3.

Following H_2_O_2_ stimulation, HDF cells in the MC group exhibited a marked reduction in the activities of SOD (Figure 7A), CAT (Figure 7C), and GSH-Px (Figure 7D), accompanied by an increase in MDA content (Figure 7B), indicating successful induction of oxidative stress. Treatment with Free-HC partially restored antioxidant enzyme levels and reduced MDA accumulation relative to the MC group. In contrast, HC-NLPs produced a more pronounced protective effect, showing higher SOD, CAT, and GSH-Px activities and a lower MDA concentration than both the MC and Free-HC groups. Consistent with these biochemical changes, fluorescence imaging using DCFH-DA and Flow cytometry quantification revealed substantial intracellular ROS accumulation in the MC group (Figure 7E and Figure S3). ROS fluorescence intensity was attenuated 39.81% in the Free-HC treated cells. Notably, HC-NLPs treatment further diminished ROS levels to 61.51%, showing the lowest fluorescence intensity among all H_2_O_2_-exposed groups. Previous studies have investigated the antioxidant effects of free ginsenoside CK and HPR in exogenous cellular senescence, reporting improvements of ~34.62% in MDA, ~13.33% in SOD, and ~43.33% in ROS. In contrast, the liposomal encapsulation strategy used in this study significantly enhanced these effects, with HPR-loaded HC-NLPs achieving ~53.67% improvement in MDA, ~76.31% in SOD, and ~61.52% in ROS. These results highlight the superior bioavailability provided by liposomal formulations and confirm the potent antioxidant activity of HC-NLPs [53].

Overall, these results indicate that both Free-HC and HC-NLPs alleviate H_2_O_2_-induced oxidative damage in HDF cells, with HC-NLPs exhibiting markedly stronger antioxidant efficacy.

### 3.8. Cellular Anti-Inflammatory Activity

TNF-α is a central mediator of cutaneous inflammatory responses that activates upstream signaling pathways, including NF-κB and MAPK. This activation subsequently promotes the transcription of various downstream cytokines such as IL-6, IL-8, and IL-1α. IL-6 contributes to inflammation-driven epidermal dysfunction, while IL-8 functions as a potent chemokine that recruits neutrophils to sites of injury or irritation. IL-1α acts as an early-response cytokine that amplifies local inflammatory cascades and enhances cytokine cross-activation. Together, these cytokines form a tightly interconnected network that reinforces and sustains epidermal inflammation. Thus, reductions in TNF-α, IL-1α, IL-8, and IL-6 collectively reflect effective suppression of inflammatory signaling in keratinocytes [54,55,56].

**Figure 8:**
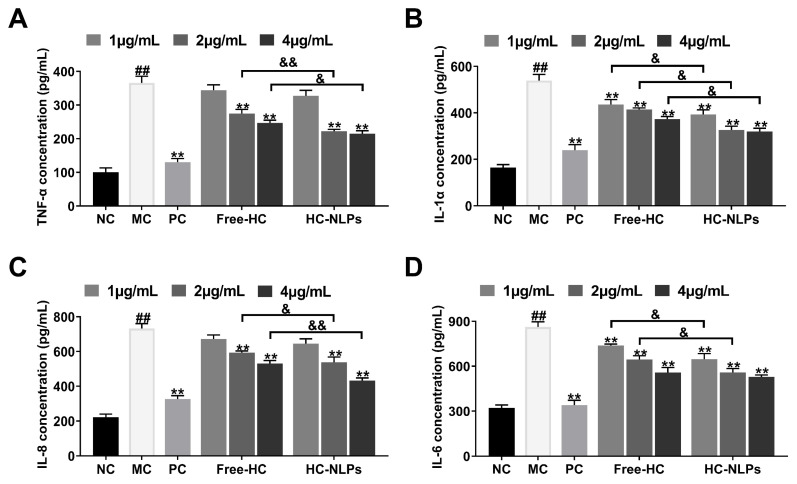
Anti-inflammatory effects of Free-HC and HC-NLPs in HDF cells. HDF cells were first stimulated with TNF-α/IFN-γ to induce inflammation and subsequently treated with Free-HC or HC-NLPs. Cytokine secretion levels of (**A**) IL-1α, (**B**) TNF-α, (**C**) IL-6, and (**D**) IL-8 were measured using ELISA kits. ^##^ *p* < 0.01 vs. NC; ** *p* < 0.01 vs. MC; ^&^ *p* < 0.05, ^&&^ *p* < 0.01. Mean ± SD, *n* = 3.

Upon stimulation with TNF-α/IFN-γ, HDF cells in the MC group exhibited a sharp elevation in TNF-α (Figure 8A), IL-1α (Figure 8B), IL-8 (Figure 8C), and IL-6 (Figure 8D) secretion, confirming the successful establishment of an inflammation model. Treatment with Free-HC attenuated this cytokine overexpression, indicating a partial anti-inflammatory effect. In contrast, HC-NLPs produced a substantially stronger inhibitory response. Across all four cytokines measured, HC-NLPs treatment resulted in markedly lower secretion levels compared with both the MC and Free-HC groups. Previous studies have compared the antioxidant capacity of five retinoid derivatives, including HPR. In that work, free HPR improved oxidative stress markers in model cells, with IL-6 and TNF-α recovery rates of ~5.26% and ~15.15%, respectively [57]. In contrast, the present study demonstrates that liposomal encapsulation significantly enhanced anti-inflammatory effects, yielding improvements of ~38.51% for IL-6 and ~41.53% for TNF-α. Furthermore, the current results show robust suppression across multiple cytokines, highlighting the ability of nanoliposomes to modulate the inflammatory cascade more comprehensively. This enhanced efficacy is particularly relevant for anti-aging treatments, where chronic low-grade inflammation contributes to skin aging. By achieving stronger and more uniform cytokine downregulation, these findings not only confirm the superior anti-inflammatory activity of HC-NLPs compared with Free-HC, but also emphasize the broader implications of liposomal encapsulation in topical applications.

### 3.9. Cellular Anti-Aging Activity

Ultraviolet A (UVA) is a major extrinsic factor driving photoaging, primarily mediated through the overproduction of ROS. Excess ROS activates MAPK and Activator Protein 1 (AP-1) signaling pathways, leading to the upregulation of matrix metalloproteinases such as MMP-1 and MMP-3. MMP-1 initiates Collagen I degradation, while MMP-3 further cleaves structural components of the extracellular matrix, thereby jointly accelerating dermal breakdown. Concurrently, UVA suppresses the synthesis of Collagen I and Collagen IV, two essential fibrillar Collagens responsible for maintaining skin tensile strength and structural integrity. Thus, the reductions in MMP-1/MMP-3 and the restoration of Collagen I/IV production are key indicators of anti-photoaging efficacy [58,59,60,61].

**Figure 9:**
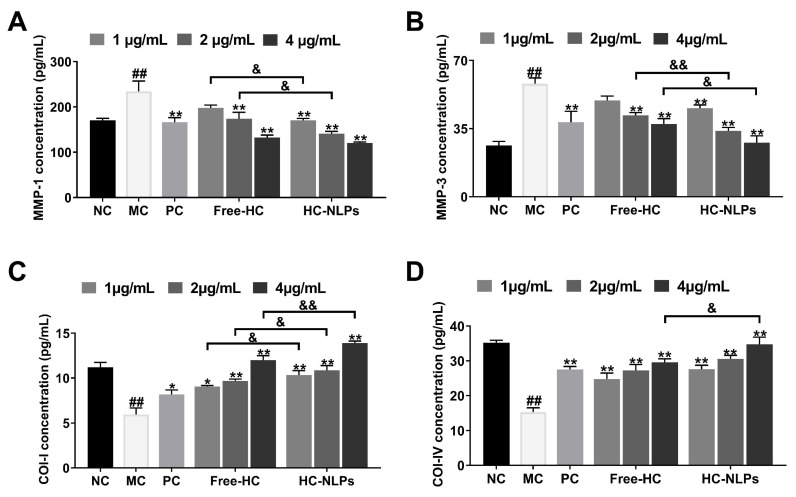
Anti-photoaging effects of Free-HC and HC-NLPs in HDF cells. HDF cells were exposed to UVA to induce photoaging-like damage, and the levels of (**A**) MMP-1 and (**B**) MMP-3 as well as (**C**) Col-I and (**D**) Col-IV were measured to evaluate extracellular matrix degradation and collagen preservation. ^##^ *p* < 0.01 vs. NC; * *p*< 0.05, ** *p* < 0.01 vs. MC; ^&^ *p* < 0.05, ^&&^ *p* < 0.01. Mean ± SD, *n* = 3.

Following UVA exposure, HDF cells in the MC group showed a marked elevation in MMP-1 (Figure 9A) and MMP-3 (Figure 9B) secretion, accompanied by a substantial reduction in Col-I and Col-IV levels, confirming successful induction of photoaging-like cellular damage. Treatment with Free-HC partially mitigated these changes, demonstrating its inherent anti-photoaging activity. HC-NLPs, however, exhibited a more pronounced protective effect. At a concentration of 4 μg/mL, HC-NLPs more effectively suppressed UVA-induced upregulation of MMP-1 (8.83%) and MMP-3 (25.62%) compared with Free-HC. In parallel, HC-NLPs significantly enhanced the recovery of Col-I (15.82%, Figure 9C) and Col-IV (17.7%, Figure 9D) compared with Free-HC. Notably, at its highest concentration, HC-NLPs produced an anti-aging effect superior to that of the positive control vitamin C. Compared with the PC group, HC-NLPs exhibited 27.49% and 27.5% stronger inhibition of MMP-1 and MMP-3, respectively, and promoted Col-I and Col-IV levels by 70.25% and 26.25%, respectively.

Taken together, these results indicate that both Free-HC and HC-NLPs confer protection against UVA-induced dermal aging, with HC-NLPs demonstrating superior efficacy in regulating extracellular matrix degradation and collagen preservation. The consistently superior performance of HC-NLPs over the Free-HC combination across various cellular models (proliferation, glycation, oxidation, and inflammation) highlights the strategic advantage of this co-delivery system. This enhancement is attributed to two primary factors: (1) the NLPs carrier significantly improves the cellular internalization of the payloads, as evidenced in the uptake study (Figure 4); and (2) the complementary action of HPR and carnosine. While HPR primarily modulates retinoid-responsive pathways for collagen renewal, carnosine provides a robust defense against oxidative and glycative damage. By integrating these two actives into a single platform, the HC-NLPs overcome their inherent solubility differences and ensure their synchronized delivery to target cells, thereby providing a more comprehensive intervention against multifaceted skin aging processes. Although formal synergistic modeling was not within the scope of this study, these integrated functional benefits demonstrate that the NLPs co-delivery strategy is a highly effective approach for advanced dermocosmetic applications.

### 3.10. Anti-Aging Effects in 3D Skin Model

**Figure 10:**
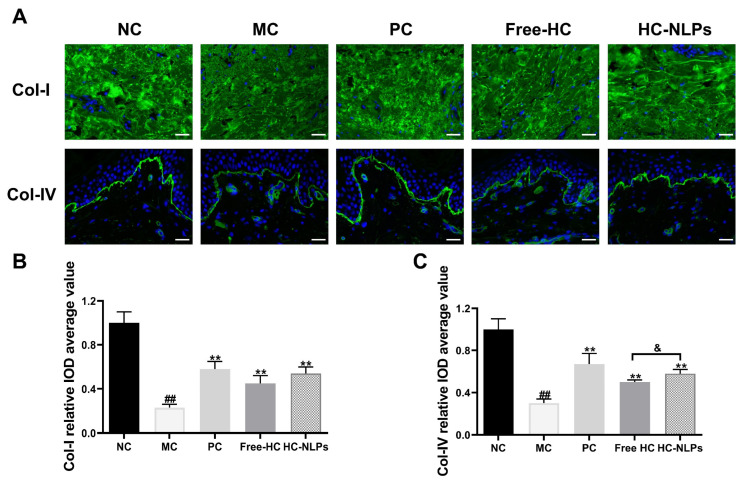
Anti-photoaging effects of Free-HC or HC-NLPs in an ex vivo skin model. ex vivo skin models were exposed to combined UVA and UVB irradiation to induce photoaging, and (**A**) Representative immunofluorescence images of Col-I and Col-IV, and quantitative analysis of the fluorescence intensity of (**B**) Col-I and (**C**) Col-IV, following treatment with Free-HC or HC-NLPs. ^##^ *p* < 0.01 vs. NC; ** *p* < 0.01 vs. MC; ^&^ *p* < 0.05. Mean ± SD, *n* = 3. Scale bar: 50 μm.

The anti-aging efficacy of Free-HC or HC-NLPs was further assessed using an ex vivo human skin model that maintains the epidermal, dermal, and stratum corneum architecture. As shown in Figure 10, exposure of the ex vivo human skin tissues to combined UVA and UVB irradiation resulted in a marked reduction in Col-I and Col-IV fluorescence intensity, reflecting substantial extracellular matrix degradation and basement membrane impairment. Compared with the MC group, both the Free-HC and HC-NLPs treatments restored the expression of Col-I and Col-IV to varying extents, increasing their levels by 95.65% and 134.78% (Col-I), and by 66.67% and 93.33% (Col-IV), respectively. Thus, compared with Free-HC, HC-NLPs induced a stronger recovery of collagen expression, yielding a denser and more continuous distribution of Col-I and Col-IV throughout the dermal and epidermal regions. Moreover, the restorative effect observed with HC-NLPs was comparable to the improvement produced by the positive control formulation containing VC and VE.

Collectively, these findings demonstrate that HC-NLPs provide superior protection against UV-induced structural aging of human skin, effectively preserving and restoring key collagen components in the 3D skin model.

### 3.11. Antioxidant Activity in Zebrafish Model

**Figure 11:**
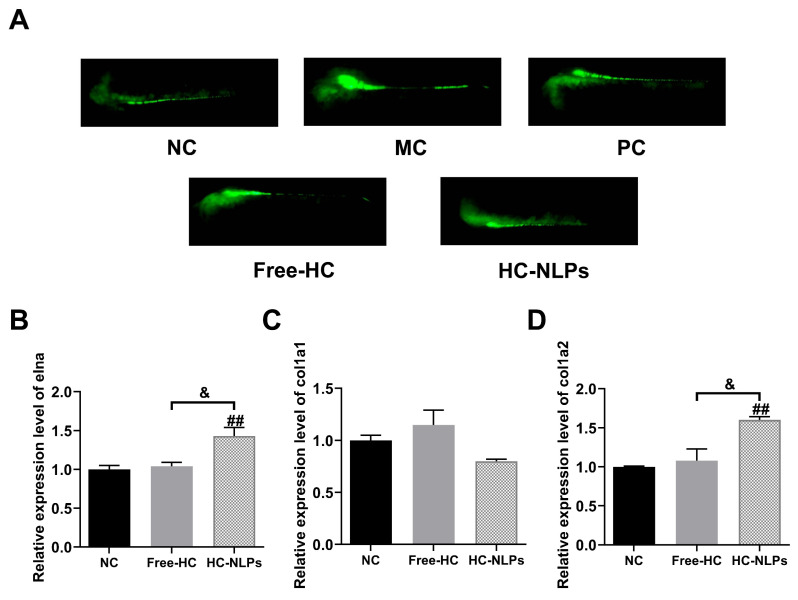
Antioxidant and ECM-protective effects of Free-HC or HC-NLPs in the hydroxyacetone-induced oxidative stress zebrafish model. (**A**) Representative fluorescence images of ROS in zebrafish. Expression of ECM-related genes, (**B**) elna, (**C**) col1a1, and (**D**) col1a2, was measured to evaluate elastin and type I collagen transcriptional regulation under oxidative stress conditions. ^##^ *p* < 0.01 vs. NC; ^&^ *p* < 0.05 vs. Free-HC; Mean ± SD, *n* = 10.

Following hydroxyacetone exposure, zebrafish embryos in the MC group displayed a conspicuous accumulation of intracellular ROS, characterized by a strong fluorescent signal, whereas only minimal fluorescence was observed in the NC group (Figure 11A). This pronounced contrast indicates severe oxidative imbalance induced by hydroxyacetone treatment. Administration of Free-HC or HC-NLPs markedly attenuated ROS-associated fluorescence in embryos. MFI analysis revealed that Free-HC decreased ROS levels by 24.97% relative to the MC group, while HC-NLPs produced a more pronounced reduction of 35.84% (Figure 11A). Both treatments significantly lowered ROS accumulation compared with the MC group (*p* < 0.01), with HC-NLPs exhibiting a significantly stronger antioxidative effect than Free-HC at the same dose (*p* < 0.01). These findings indicate that nanoliposomal encapsulation markedly enhances the antioxidative efficacy of HC, enabling more effective alleviation of hydroxyacetone-induced oxidative damage in zebrafish embryos.

Elna, col1a1, and col1a2 are key extracellular matrix (ECM)-related genes involved in maintaining structural integrity and tissue resilience. elna encodes elastin, which contributes to elastic fiber formation and provides flexibility and mechanical support. col1a1 and col1a2 encode the two major α-chains required for proper assembly of type I collagen, the most abundant structural collagen responsible for ECM strength and stability. Under oxidative stress, suppression of these genes reflects impaired ECM homeostasis and degradation of elastic and collagenous components. Therefore, upregulation of elna, col1a1, and col1a2 indicates enhanced ECM repair, restoration of collagen fiber architecture, and improved tissue resilience following treatment. Compared with the NC group, both Free-HC and HC-NLPs treatment upregulated the expression of ECM-related genes in zebrafish embryos (Figure 11B–D). Notably, HC-NLPs elicited a stronger stimulatory effect than Free-HC for certain targets: elna expression was increased by 37.5% relative to Free-HC, and col1a2 expression was 48.14% higher in the HC-NLPs group than the Free-HC group. In contrast, no significant difference was observed between HC-NLPs and Free-HC groups in col1a1 expression. These results indicate that HC-NLPs more effectively promote elastin and type I collagen α2 chain transcription than the free formulation, while the effect on the col1a1 transcript is comparable to that of the free formulations.

Taken together, the results suggest a clear relationship between the physicochemical properties of HC-NLPs and their biological performance. The nanoscale size and lipid composition of the system likely facilitate its interaction with the stratum corneum and improve epidermal penetration, thereby enhancing cellular uptake and intracellular availability of the active compounds. This improved delivery is associated with the enhanced antioxidant, anti-glycation, anti-inflammatory, and extracellular matrix–protective effects observed in vitro. Importantly, these cellular-level effects are also reflected in the improved anti-aging outcomes observed in the 3D ex vivo skin model and the zebrafish model. Overall, these findings indicate that NLPs encapsulation can enhance both delivery efficiency and biological activity, contributing to improved performance across different biological levels.

## 4. Conclusions

In this study, HPR and CA were successfully co-encapsulated into nanoliposomes (HC-NLPs) to enhance their stability and skin delivery. HC-NLPs demonstrated markedly improved epidermal/dermal delivery and cellular uptake compared to the free combination. This enhanced delivery translated into superior multi-functional bioactivity. HC-NLPs potently promoted cell proliferation and migration, alleviated oxidative stress and inflammation, inhibited glycation, and attenuated cellular senescence. These effects were further validated in a 3D skin model, where HC-NLPs enhanced collagen deposition, and in a zebrafish model, where they reduced ROS and up-regulated extracellular-matrix gene expression. Collectively, the nano-liposomal strategy effectively overcomes the inherent limitations of HPR and CA, complementarily amplifying their anti-aging, antioxidant, anti-inflammatory, and anti-glycation activities. While this work validates efficacy at the functional and phenotypic levels, further research is required to elucidate the specific upstream signaling cascades, conduct formal synergistic modeling, assess release kinetics under skin-mimetic acidic conditions, and evaluate the system’s photostability under UV radiation [62]. HC-NLPs represent a promising and effective topical delivery platform for advanced dermocosmetic applications.

## Figures and Tables

**Table 1:** Primers used for RT-PCR.

Gene	Primer 5′-3′
GAPDH-R	GGAAGCTTGTCATCAATGGAAATC
GAPDH-F	TGATGACCCTTTTGGCTCCC
RAGE-R	CTCAGGACCAGGGAACCTACAG
RAGE-F	CGCCTTTGCCACAAGATGAC
NADPH-R	TTCTAAATGGCACAGCCTTCGT
NADPH-F	GGTTGAATCACATTGAATCGCA

**Table 2:** EE and DLE of HC-NLPs.

Active Ingredient	EE	DLE
HPR	97.3 ± 0.8	1.60 ± 0.3
CA	71.4 ± 0.4	0.70 ± 0.1

## Data Availability

Data available on request from the authors.

## Supplementary Materials

The following supporting information can be downloaded at: https://www.mdpi.com/article/10.3390/pharmaceutics18040454/s1, Table S1: Concentration of HPR during storage of HC-NLPs, Table S2: Particle size and PDI of HC-NLPs during storage under various conditions; Table S3: CAM Stimulation Rating Scale; Figure S1. (A) MFI and (B) penetration depth of Free-RhoB or RhoB-NLPs at different times after skin treatment. ^&^ *p* < 0.05, ^&&^ *p* < 0.01 vs. Free-HC. Mean ± SD, *n* = 3; Figure S2. Analysis of cellular uptake by flow cytometry in HDF cells treated with Free-RhoB or RhoB-NLPs. ^&^ *p* < 0.05 vs. Free-RhoB. Mean ± SD, *n* = 3; Figure S3. Antioxidant effects of Free-HC and HC-NLPs in HDF cells. Quantitative analysis of intracellular ROS levels by flow cytometry. ^##^ *p* < 0.01 vs. NC; ** *p* < 0.01 vs. MC; ^&^ *p* < 0.05 vs. Free-HC. Mean ± SD, *n* = 3; Figure S4. Antioxidant and ECM-protective effects of Free-HC or HC-NLPs in the hydroxyacetone-induced oxidative stress zebrafish model. The quantitative analysis of fluorescence images of ROS in zebrafish. ^##^ *p* < 0.01 vs. NC; ** *p* < 0.01 vs. MC; ^&^ *p* < 0.05 vs. Free-HC. Mean ± SD, *n* = 3.
