# Supplementary material for: A Nanoliposome Platform Co-Delivery of Hydroxypinacolone Retinoate and Carnosine for Enhanced Epidermal/Dermal Delivery and Multi-Functional Anti-Aging Efficacy
Source: Pharmaceutics. 2026 Apr 8;18(4):454. doi: 10.3390/pharmaceutics18040454 (PMC13119431; doi:10.3390/pharmaceutics18040454)
Supplement: Supplementary file 1 [file pharmaceutics-18-00454-s001.zip › pharmaceutics-4200574-supplementary.pdf]

Supplemental material

A Nanoliposome Platform Co-Delivery of Hydroxypinacolone Retinoate and Carnosine for Enhanced Epidermal/Dermal Delivery and Multi-Functional Anti-Aging Efficacy

Siyuan Chen [1,†], Lihao Gu [2,†], Ruili Zhao [2], Lihua Zhang [2], Lina Yao [2], Jingning Shen [2], Dan Luo [3,4], Xi Wang [4], Dan Chen [4], Si Zhao [4], Hong Zhou [5,*] and Wei Liu [3,6,*]

1 Research Institute for Biomaterials, Tech Institute for Advanced Materials Bioinspired Biomedical Materials & Devices Center, College of Materials Science and Engineering, Jiangsu Collaborative Innovation Center for Advanced Inorganic Function Composites, Suqian Advanced Materials Industry Technology Innovation Center, Nanjing Tech University, Nanjing 211816, China
2 Osman Biological Co., Ltd., Huzhou 313200, China
3 National Engineering Research Center for Nanomedicine, Huazhong University of Science and Technology, Wuhan 430075, China
4 Wuhan Bestcarrier Biotechnology Co., Ltd., Wuhan 430074, China
5 USDA/ARS Children's Nutrition Research Center, Department of Pediatrics, Baylor College of Medicine, Houston 77030, TX, USA
6 College of Life Science and Technology, Huazhong University of Science and Technology, Wuhan 430074, China
* Correspondence: hong.zhou@bcm.edu (H.Z.); wliu@hust.edu.cn (W.L.)
† These authors contributed equally to this work.

.

Table S1. Concentration of HPR during storage of HC-NLPs

Group (Concentration of HPR)	0 day	30 day	60 day
RT	2%	1.84%	1.50%
Light	2%	1.79%	1.30%
-20℃	2%	1.79%	1.70%
4℃	2%	1.96%	1.65%
45℃	2%	1.95%	1.28%

Table S2. Particle size and PDI of HC-NLPs during storage under various conditions.

	0 day		30 day		60 day	
	Z-Average Size	PDI	Z-Average Size	PDI	Z-Average Size	PDI
-20 ℃	107.9	0.128	108.0	0.130	108.0	0.134
4 ℃	108.3	0.127	108.5	0.129	109.0	0.136
RT	110.7	0.118	110.8	0.121	111.7	0.125
45 ℃	110.3	0.111	110.4	0.112	114.4	0.131
Light	108.9	0.118	112.1	0.127	116.7	0.136

Table S3 CAM Stimulation Rating Scale

Number	Sample	IS HET
1	Normal Saline	0.07
2	0.1% NaOH	13.27
3	Free-HC	0.07
4	HC-NLPs	0.07

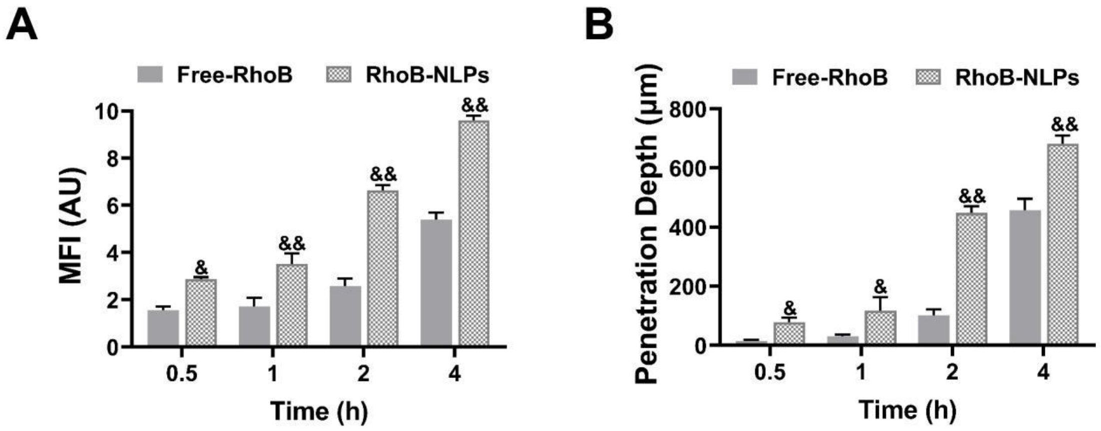

Figure S1. (A) MFI and (B) penetration depth of Free-RhoB or RhoB-NLPs at different times after skin treatment. $^{\&}p < 0.05$, $^{\&\&}p < 0.01$ vs Free-HC. Mean ± SD, n=3.

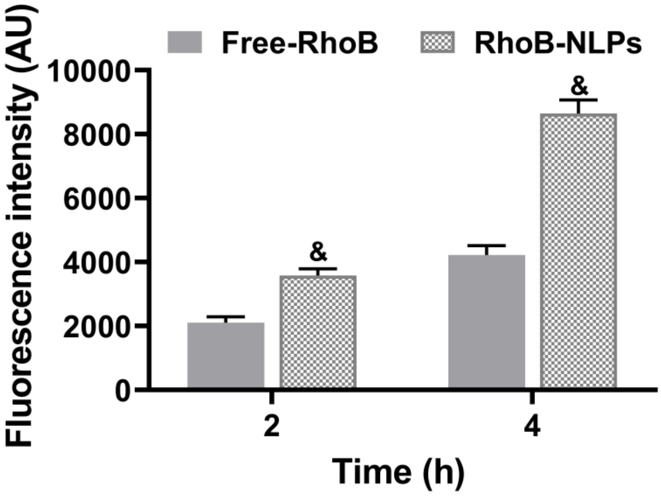

Figure S2. Analysis of cellular uptake by flow cytometry in HDF cells treated with Free-RhoB or RhoB-NLPs. $^{\&}p < 0.05$ vs Free-RhoB. Mean ± SD, $n = 3$.

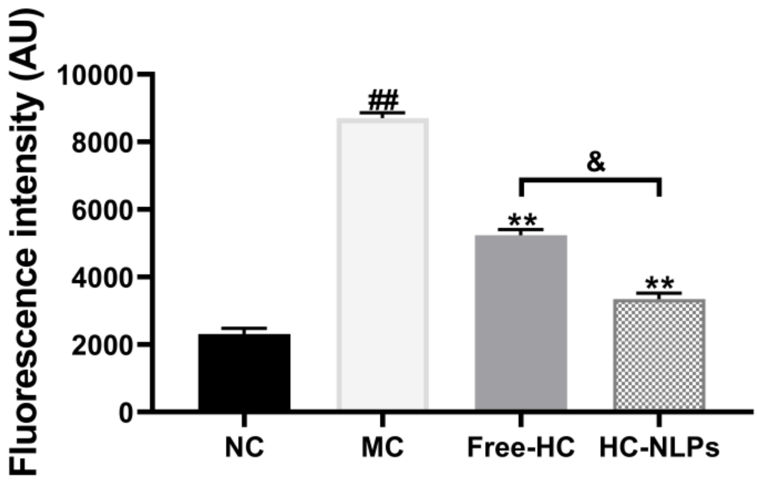

Figure S3. Antioxidant effects of Free-HC and HC-NLPs in HDF cells. Quantitative analysis of intracellular ROS levels by flow cytometry. ##p < 0.01 vs NC; **p < 0.01 vs MC; &p < 0.05 vs Free-HC. Mean ± SD, n = 3.

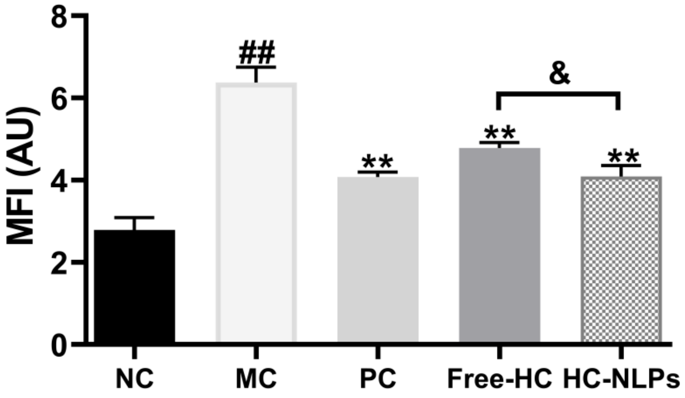

Figure S4. Antioxidant and ECM-protective effects of Free-HC or HC-NLPs in the hydroxyacetone-induced oxidative stress zebrafish model. The quantitative analysis of fluorescence images of ROS in zebrafish. $^{\#\#}p < 0.01$ vs NC; $^{**}p < 0.01$ vs MC; $^{\&}p < 0.05$ vs Free-HC. Mean ± SD, $n = 3$.
